# Changes in the serum metabolomics of polycystic ovary syndrome before and after compound oral contraceptive treatment

**DOI:** 10.3389/fendo.2024.1354214

**Published:** 2024-06-14

**Authors:** Ting Zhao, Xiao Xiao, Lingchuan Li, Jing Zhu, Wenli He, Qiong Zhang, Jiaqi Wu, Xiaomei Wu, Tao Yuan

**Affiliations:** ^1^ Department of Gynecology, The First People’s Hospital of Yunnan Province, Kunming, China; ^2^ Department of Gynecology, The Affiliated Hospital of Kunming University of Science and Technology, Kunming, China

**Keywords:** metabolomics analysis, ultra-performance liquid chromatography-high-resolution mass spectrometry, polycystic ovary syndrome, serum metabolites, COC

## Abstract

**Background:**

Polycystic ovary syndrome (PCOS) is both a common endocrine syndrome and a metabolic disorder that results in harm to the reproductive system and whole-body metabolism. This study aimed to investigate differences in the serum metabolic profiles of patients with PCOS compared with healthy controls, in addition to investigating the effects of compound oral contraceptive (COC) treatment in patients with PCOS.

**Materials and methods:**

50 patients with PCOS and 50 sex-matched healthy controls were recruited. Patients with PCOS received three cycles of self-administered COC treatment. Clinical characteristics were recorded, and the laboratory biochemical data were detected. We utilized ultra-performance liquid chromatography–high-resolution mass spectrometry to study the serum metabolic changes between patients with PCOS, patients with PCOS following COC treatment, and healthy controls.

**Result:**

Patients with PCOS who received COC treatment showed significant improvements in serum sex hormone levels, a reduction in luteinising hormone levels, and a significant reduction in the levels of biologically active free testosterone in the blood. Differential metabolite correlation analysis revealed differences between PCOS and healthy control groups in N-tetradecanamide, hexadecanamide, 10E,12Z-octadecadienoic acid, and 13-HOTrE(r); after 3 months of COC treatment, there were significant differences in benzoic acid, organic acid, and phenolamides. Using gas chromatography–mass spectrometry to analyse blood serum in each group, the characteristic changes in PCOS were metabolic disorders of amino acids, carbohydrates, and purines, with significant changes in the levels of total cholesterol, uric acid, phenylalanine, aspartic acid, and glutamate.

**Conclusion:**

Following COC treatment, improvements in sex hormone levels, endocrine factor levels, and metabolic levels were better than in the group of PCOS patients receiving no COC treatment, indicating that COC treatment for PCOS could effectively regulate the levels of sex hormones, endocrine factors, and serum metabolic profiles.

## 1 Introduction

Polycystic ovary syndrome (PCOS) is the most common endocrine disorder worldwide, affecting approximately 15–20% of women of childbearing age (1). Research has shown that patients with PCOS have a significantly higher risk of infertility, pregnancy complications, and miscarriage than healthy women (2). In addition to reproductive dysfunction, PCOS is closely related to metabolic abnormalities, such as insulin resistance (IR), hyperinsulinemia, cardiovascular and cerebrovascular diseases, obesity, and lipid metabolism disorders, which can seriously affect quality of life. However, metabolism is regulated by multiple factors, such as changes in the gut microbiome and the involvement of inflammatory mediators (3, 4). PCOS is a complex and systemic neuroendocrine metabolic network disorder that is mainly characterized by gonadal axis dysfunction, seriously endangering women’s health.

At present, due to the unclear aetiology and pathogenesis of PCOS, there is no radical cure for the disease. The treatment methods are symptomatic, and specific treatment plans must be formulated based on the patient’s age, treatment needs, and specific manifestations of the disease (5, 6). In patients with PCOS who present with clinically high androgen levels or abnormal biochemical test results, androgen levels can be reduced using medication, including compound oral contraceptives (COCs), adrenocortical hormones, and spironolactone. The preferred drug is COCs. For adolescent patients and those of reproductive age with PCOS, COC treatment is preferred for hyperandrogenism (HA) and clinical manifestations of high androgen levels (7).

Metabolomics is the qualitative and quantitative analysis of metabolites in the body, which is used to determine the relative relationship between specific metabolites and diseases and their phenotypic changes; therefore, it can quickly and sensitively reflect changes in the overall function of the biological system (8, 9). Compared to genomics, transcriptomics, and proteomics, metabolomics can more intuitively display changes in the body and is closely related to disease phenotypes. The development and transformation applications of metabolomics technology have provided a new platform for constructing diagnostic and treatment strategies for PCOS, in addition to exploring its pathogenesis. The untargeted metabolomics approach, known as metabolic fingerprinting, mainly focuses on the identification and quantification of as many low-molecular-weight compounds present in tested samples as possible (10).

In this study, using metabolomics methods and ultra-high performance liquid chromatography–high-resolution mass spectrometry (UPLC–HRMS), we aimed to investigate changes in the metabolic fingerprints of patients with PCOS before and after treatment with COCs in order to identify potential prognostic and diagnostic metabolic markers of the disease.

## 2 Methods

### 2.1 Study participants

All patients with PCOS and healthy controls were recruited from the First People’s Hospital of Yunnan Province (Kunming, China). This study was approved by the First People’s Hospital of Yunnan Province. Written informed consent was obtained from all participants before study inclusion. According to the Rotterdam criteria (2003), PCOS can be diagnosed if two of three criteria are present after excluding congenital adrenal hyperplasia, Cushing’s syndrome, androgen-secreting tumours, or other related disorders. The three criteria are as follows: (1) oligo- and/or anovulation; (2) clinical and/or biochemical signs of HA (clinical manifestations of HA include the presence of acne, hirsutism, and androgenic alopecia); and (3) polycystic ovaries observed via ultrasound examination, which includes the presence of 12 or more follicles in each ovary measuring 2–9 mm in diameter and/or an ovarian volume > 10 cm^3^.

The inclusion criteria for the subjects were as follows: (1) To prevent metabolomic differences among patients with different types of PCOS, meet the Rotterdam diagnostic criteria of PCOS phenotype B with hyperandrogenism and ovulatory dysfunction; (2) body mass index (BMI) 18~24 kg/m2; (3) age between 18 and 40 years; (4) no medication that affects insulin sensitivity or ovarian function within the first three months of the trial.

In order to prevent metabolomics differences between different types of PCOS patients, 50 B-type PCOS patients were uniformly selected. These 50 B-PCOS started treatment with COC from the 5th day of menstruation or drug withdrawal bleeding and took one tablet per day for 21 consecutive days. After stopping the medication, the next cycle started from the 5th day of withdrawal bleeding, totalling three cycles. Changes between basal and post treatment results from hormonal and metabolomic data were assessed.

### 2.2 Collection and pre-treatment of serum samples

Each sample (100 μl) was transferred to a 2-ml Eppendorf tube and resuspended in pre-cooled methanol at -20°C through an eddy current for 60 s. The blood sample was then incubated on ice for 15 min, centrifuged at 4°C for 20 min at 12000 rpm, and the supernatant was transferred to a new centrifuge tube. Liquid chromatography–mass spectrometry (LC–MS)-grade water was used to dilute part of the supernatant to the final concentration of 53% methanol. The samples were then transferred to a fresh Eppendorf tube and centrifuged at 15000 × g for 20 min. The initial samples for LC–MS analysis were obtained using 0.22-µm membrane filtration and were injected into the LC–MS/MS system for analysis (11).

### 2.3 HPLC–MS/MS analysis

LC–MS/MS analyses were performed using an ExionLC™ AD system (SCIEX) coupled with a QTRAP® 6500+ mass spectrometer (SCIEX) from Novogene Co., Ltd. (Beijing, China). Samples were injected onto Xselect HSS T3 (2.1 × 150 mm, 2.5 μm) using a 20-min linear gradient at a flow rate of 0.4 ml/min for the positive/negative polarity mode. The eluents were eluent A (0.1% formic acid–water) and eluent B (0.1% formic acid–acetonitrile) (12). The solvent gradient was set as follows: 2% B, 2 min; 2–100% B, 15.0 min; 100% B, 17.0 min; 100–2% B, 17.1 min; and 2% B, 20 min. The QTRAP® 6500+ mass spectrometer was operated in positive polarity mode with a (curtain gas, 35 psi; collision gas, medium; IonSpray voltage, 5500 V; temperature, 550 °C; ion source gas 1, 60 psi; ion source gas 2, 60 psi). The QTRAP® 6500+ mass spectrometer was operated in negative polarity mode (curtain gas, 35psi; collision gas, medium; IonSpray voltage, -4500V; temperature, 550°C; ion source gas 1, 60psi; ion source gas 2, 60psi).

### 2.4 Metabolite identification and quantification

The detection of the experimental samples using multiple reactions monitoring (MRM) was based on the Novogene in-house database. Q3 was used for the metabolite quantification. The Q1, Q3, RT (retention time), DP (declustering potential) and CE (collision energy) were used in metabolite identification. The data files generated by HPLC–MS/MS were processed using the SCIEX OS version 1.4 to integrate and correct the peak. The main parameters were set as follows: minimum peak height, 500; signal/noise ratio, 5; Gaussian smooth width, 6; the area of each peak represents the relative content of the corresponding substance.

### 2.5 Data analysis

R software (version 3.3; Boston, MA, USA) and SPSS (version 20.0; IBM Corp., Armonk, NY, USA) were used for all bioinformatics and statistical analyses. The data are expressed as mean ± standard deviation. Metabolites were annotated using the Kyoto Encyclopaedia of Genes and Genomes (KEGG), the human metabolome database (HMDB), and the lipid map database. Principal component analysis (PCA) and partial least squares discriminant analysis (PLS-DA) were performed using metaX (13). Univariate analysis (t-test) was used to calculate statistical significance (p-values). Metabolites with variable importance in projection (VIP) >1, p<0.05, and fold change (FC) ≥2 or ≤0.5 were considered differential metabolites. ggplot2 in R was used to filter the metabolites of interest according to Log2 (FC) and -log10 (p-value) of the metabolites.

For clustering heat maps, the Z-scores of different metabolite intensity regions were used to normalize the data, and the Pheatmap package in R was used to plot. The cor.mtest function in R was used to calculate statistically significant correlations between different metabolites. Statistical significance was set to p<0.05 and the corrplot package in R were used to plot the correlation graph. The KEGG database was used to analyse the functions and metabolic pathways of these metabolites. The metabolic pathways of different metabolites were deemed to be enriched when the ratio met x/n>y/n. When the p-value of the metabolic pathway was <0.05, the metabolic pathway was considered to be statistically significant.

The SPSS Statistics 21 program was used to analyse the data. Descriptive statistics were used to describe continuous and categorical variables. Independent Samples T-test was run to determine the difference in PCOS and control, PCOS-treatment and control. Paired samples T-test was run to determine the difference in PCOS and PCOS-treatment after treatment with COC.

## 3 Results

### 3.1 Changes in serum indicator levels in patients with PCOS before and after COC treatment

The clinical characteristics and biochemical data of the study participants were collected and analysed (**Table 1**). The study participants included 50 healthy controls and 50 women with PCOS. There were no statistically significant differences in age or body mass index between the two groups (p>0.05). The levels of fasting glucose, luteinising hormone (LH), free testosterone, triglycerides, low-density lipoprotein cholesterol and the LH/follicle-stimulating hormone ratio were significantly higher in patients with PCOS than in controls, and prolactin and high-density lipoprotein cholesterol levels were significantly lower in patients with PCOS than in controls (p<0.05). However, after 3 months of COC treatment, the improvements in sex hormone levels in the PCOS group were better than before treatment, with significant improvements in LH, the LH/FSH ratio, oestrogen, free testosterone and triglycerides.

**Table 1:** Changes in serum hormone levels before and after compound oral contraceptive (COC) treatment.

	PCOS (n=50)	PCOS-treatment (n=50)	Control (n=50)	p1-value (1VS 3)	p2-value (1VS 2)	p3-value (2VS 3)
Age (years)	25.59 ± 4.78	25.89 ± 4.14	26.79 ± 5.52	0.320	0.315	0.234
BMI (kg/m^2^)	24.01 ± 3.15	23.25 ± 4.12	22.91 ± 3.15	0.064	0.213	0.075
FSH (IU/L)	6.23 ± 2.16	5.16 ± 3.45	5.11 ± 2.05	0.078	0.062	0.074
LH (IU/L)	13.05 ± 5.26	8.05 ± 4.13	7.89 ± 3.15	0.002*	0.005*	0.411
LH/FSH ratio	2.26 ± 1.45	1.56 ± 0.81	1.54 ± 0.83	0.004*	0.011*	0.073
PRL (ng/mL)	14.56 ± 7.16	14.06 ± 6.59	13.02 ± 6.28	0.060	0.067	0.325
E2 (pmol/L)	176.56 ± 56.85	152 ± 57.26	148.56 ± 61.23	0.042*	0.05*	0.345
Free Testosterone (nmol/l)	2.05 ± 1.52	0.91 ± 0.26	0.81 ± 0.49	0.004*	0.005*	0.215
TG	1.79 ± 1.51	1.26 ± 1.02	1.01 ± 0.37	0.001*	0.026*	0.061
CHOL	4.66 ± 1.03	4.45 ± 1.02	4.38 ± 0.97	0.068	0.070	0.083
HDL-C	1.30 ± 0.34	1.30 ± 0.25	1.30 ± 0.20	0.818	0.936	0.921
LDL-C	2.75 ± 0.81	2.64 ± 0.85	2.57 ± 0.53	0.052	0.103	0.112
HOMA-IR	3.74± 1.49	2.46 ± 1.09	2.23 ± 0.86	0.001*	0.002*	0.852

* *p* value <0.05. the bold values meaning p<0.05.

### 3.2 Multivariate statistical analysis

**Figure 1:**
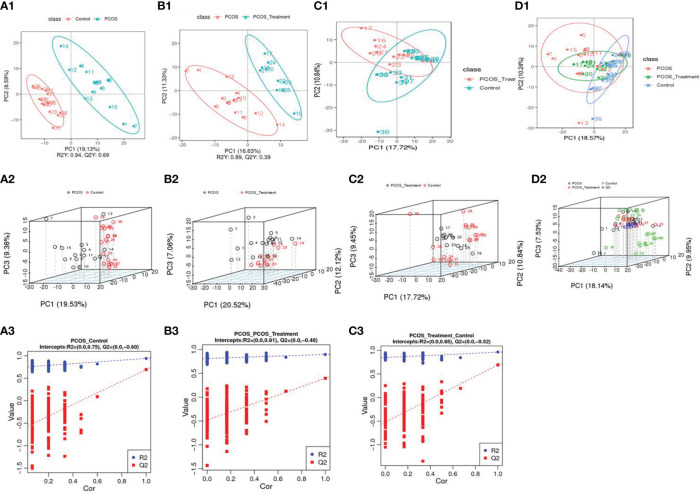
Using principal component analysis, the overall distribution trends were observed and compared between two groups of samples: **(A1–A3)** compositional differences between the polycystic ovary syndrome (PCOS) group and the healthy control group; **(A1)** partial least squares discriminant analysis (PLS-DA) score scatter plot and sorting verification chart. **(A2)** the horizontal axis (PC1) and vertical axis (PC2) represent the scores of the first- and second-ranked principal components, respectively. Scatters of different colours represent samples from different experimental groups, and ellipses represent the 95% confidence interval; **(A3)** R2Y represents the explanatory power of the model, Q2Y was used to evaluate the predictive ability of the PLS-DA model, and when R2Y is greater than Q2Y, it indicated that the model was well established; **(B1–B3)** total component difference between the PCOS group and the PCOS treatment group; **(C1–C3)** total compositional differences between POCS treatment and healthy control groups; **(D1, D2)** compositional differences among the three groups.

PCA was used to classify the original distribution of the metabolites in patients with PCOS, which generally reflects the overall metabolic differences between groups of samples and the variability between samples in the group. As shown in **Figure 1**, samples from the PCOS group could be clearly distinguished from those from healthy controls, and there was no significant difference in the serum metabolic composition between patients with PCOS treated with COC and the healthy control group.

### 3.3 LC–MS identification of significant changes in metabolites

**Figure 2:**
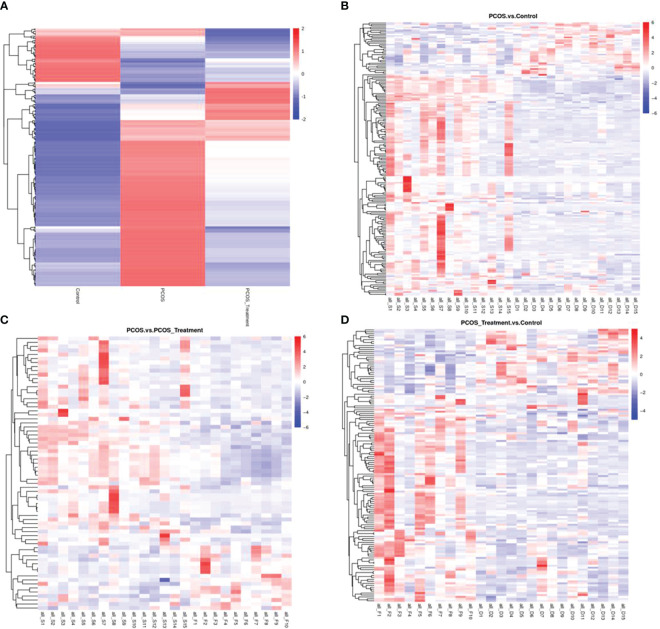
Total differential metabolite clustering heatmap and group differential metabolite clustering analysis; **(A)** cluster diagram of total differential metabolites among patients with polycystic ovary syndrome (PCOS), patients with PCOS receiving treatment, and control groups; **(B)** cluster analysis of differential metabolites between PCOS vs. control and **(C)** PCOS vs. PCOS-treatment; **(D)** cluster analysis of differential metabolites between PCOS-treatment and control groups; Vertical clustering refers to the clustering of samples, while horizontal clustering refers to the clustering of metabolites. The shorter the clustering branch, the higher the similarity. The relationship between the clustering of metabolite content between groups can be seen through horizontal comparison.

**Figure 3:**
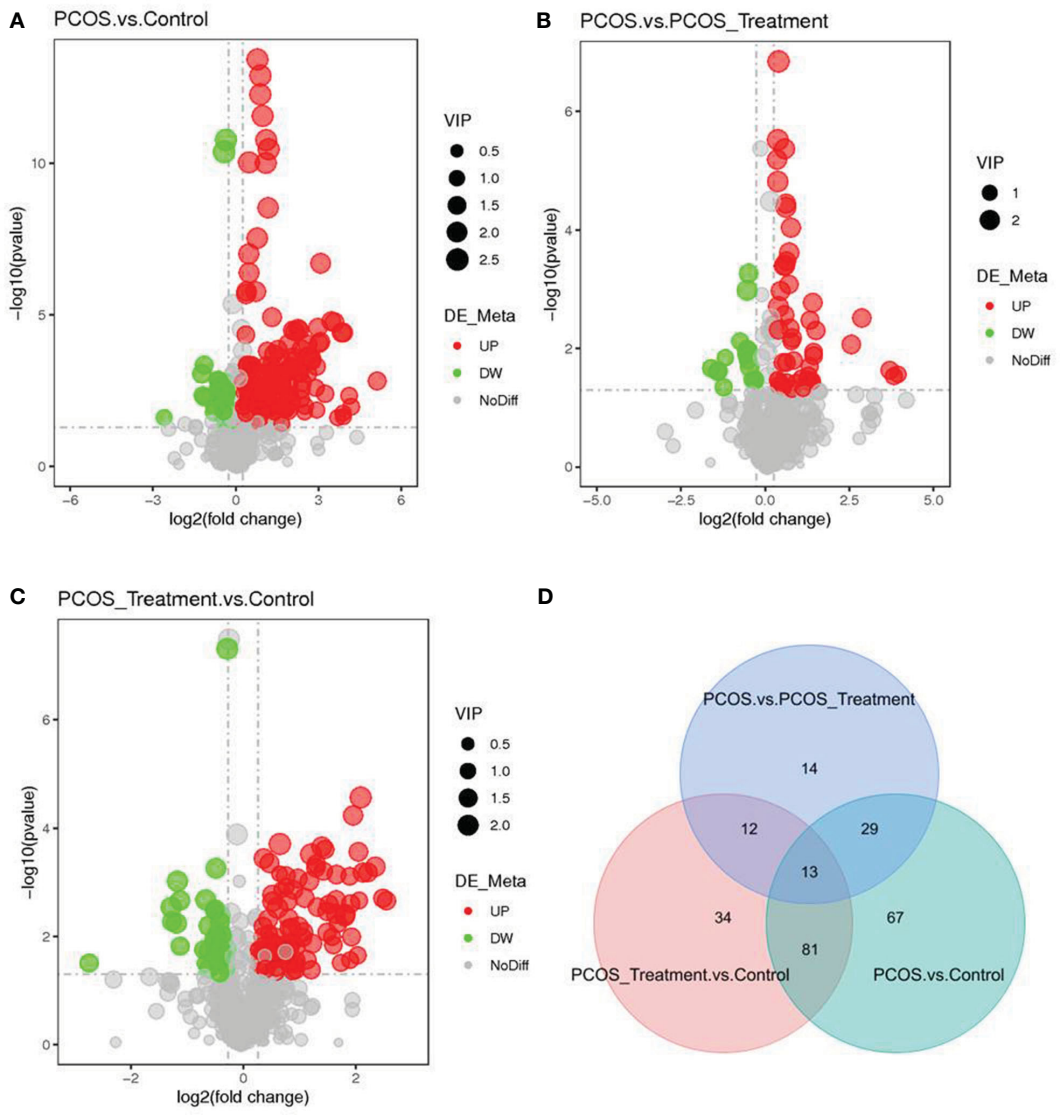
Identification of significantly altered metabolites using ultra-performance liquid chromatography–high-resolution mass spectrometry (UPLC-HRMS); A volcano plot was used to visualize differential metabolites of significance between the **(A)** polycystic ovary syndrome (PCOS) and control groups; **(B)** PCOS vs. PCOS-treatment groups; and **(C)** PCOS-treatment vs. control groups. Metabolites with false discovery rate (FDR)-adjusted p-value ≤0.05 are highlighted in red (upregulated) or green (downregulated); **(D)** clustering analysis using significant differential metabolites performed using the Student’s t-test (FDR-adjusted p-value <0.05).

As shown in **Figure 2**, compared with the control group, the metabolic components of the PCOS group showed significant differences, in addition to differences in components after COC treatment between the two groups. In this study, the metabolomic analysis of serum samples from patients with PCOS revealed 660 metabolites. A volcano plot was used to visually display the overall distribution of the differential metabolites. Each point in the volcano plot represents a metabolite; significantly upregulated metabolites are represented by red dots, and significantly downregulated metabolites are represented by green dots. As shown in **Figure 3**, when comparing patients with PCOS with controls, a total of 190 metabolites significantly differed, and these were selected as potential biomarkers of PCOS for subsequent analyses. The volcano plot showed that, compared with the control group, among these metabolites, 36 were downregulated and 154 were upregulated (**Figure 3A**). When comparing the PCOS and PCOS-treatment groups, a total of 68 significantly changed metabolites were identified; 16 were downregulated and 52 were upregulated. When comparing the PCOS-treatment and control groups, a total of 140 significantly changed metabolites were identified; 35 were downregulated and 105 were upregulated (**Figures 3B, C**).

### 3.4 Metabolite enrichment and metabolic pathway analysis

**Figure 4:**
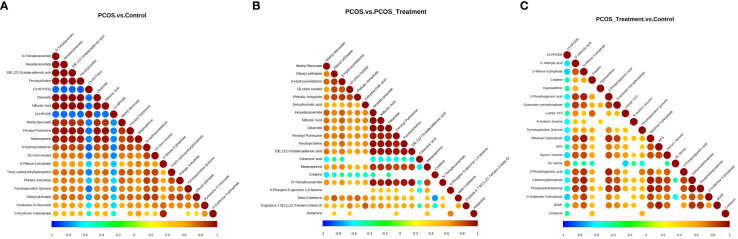
Correlation analysis of different metabolites in different groups; **(A)** analysis of differential metabolites in the polycystic ovary syndrome (PCOS) vs. control groups; **(B)** analysis of differential metabolites between PCOS and PCOS-treatment groups; **(C)** differential metabolite analysis, PCOS-treatment vs. control groups. The highest possible correlation was 1, indicating completely positive correlation (red), and the lowest possible correlation was -1, indicating completely negative correlation (blue). The parts without colour indicate a p-value>0.05. The figure shows the correlation of differential metabolites in the Top 20 sorted by p-values from smallest to largest.

The differential metabolite correlation analysis in the PCOS and healthy control groups revealed that the top 10 differential metabolic components included N-tetradecanamide, hexadecanamide, 10E,12Z-octadecadienoic acid, feruloylcholine, 13-HOTrE(r), oleamide, niflumic acid, 13-HPODE, methyl benzoate and feruloyl putrescine (**Table 2**, **Figure 4A**). After 3 months of COC treatment, significant differences in benzoic acid, organic acid, phenolamides, phenols, organoheterocyclic compounds, bile acids, fatty acyls and others were observed between the PCOS and PCOS-treatment groups (**Table 3**, **Figure 4B**). The top 10 significantly differentially expressed metabolites identified between the PCOS-treatment and control groups comprised those that were upregulated, including benzoic acid, organic acid, phenolamides, phenols, organoheterocyclic compounds, bile acids, fatty acyls and others (**Table 4**, **Figure 4C**).

**Table 2:** Differential metabolites identified between the polycystic ovary syndrome (PCOS) and control groups.

Class	Metabolite	Formula	Compound ID	p-value	ROC	Up or Down
N-tetradecanamide	Fatty acyls	C14H29NO	Com_539_pos	3.88E-14	1	up
Hexadecanamide	Fatty acyls	C16H33NO	Com_347_pos	1.32E-13	1	up
10E,12Z-octadecadienoic acid	Fatty acyls	C18H32O2	Com_414_pos	5.51E-13	1	up
Feruloylcholine	Cholines	C15H22NO4	Com_362_pos	2.82E-12	1	up
13-HOTrE(r)	Eicosanoid	C18H30O3	Com_11_neg	1.74E-11	1	down
Oleamide	Fatty acyls	C18H35NO	Com_585_pos	1.76E-11	1	up
Niflumic acid	Others	C13H9F3N2O2	Com_534_pos	3.52E-11	1	up
13-HPODE	Eicosanoid	C18H32O4	Com_246_neg	4.34E-11	1	down
Methyl benzoate	Benzoic acid	C8H8O2	Com_477_pos	9.54E-11	0.99	up
Feruloyl putrescine	Phenolamides	C14H20N2O3	Com_526_pos	9.96E-11	1	up

**Table 3:** Differential metabolites identified between the polycystic ovary syndrome (PCOS) and PCOS-treatment groups.

Class	Metabolite	Formula	Compound ID	p-value	ROC	Up or Down
Methyl benzoate	Benzoic acid	C8H8O2	Com_477_pos	1.44E-07	0.98	up
Dibutyl phthalate	Organic acid	C16H22O4	Com_452_pos	3.04E-06	0.96	up
6-Hydroxymelatonin	Phenolamides	C13H16N2O3	Com_457_pos	4.30E-06	0.953333	up
1D-chiro-inositol	Phenols	C6H12O6	Com_385_pos	6.54E-06	0.993333	up
Phthalic anhydride	Organoheterocyclic compounds	C8H4O3	Com_620_pos	1.53E-05	0.96	up
Dehydrocholic acid	Bile acids	C24H34O5	Com_344_pos	3.60E-05	0.92	up
Hexadecanamide	Fatty acyls	C16H33NO	Com_347_pos	4.16E-05	0.973333	up
Niflumic acid	Others	C13H9F3N2O2	Com_534_pos	9.20E-05	0.966667	up
Oleamide	Fatty acyls	C18H35NO	Com_585_pos	0.000242	0.96	up
Feruloyl putrescine	Phenolamides	C14H20N2O3	Com_526_pos	0.000337	0.933333	up

**Table 4:** Differential metabolites identified between the polycystic ovary syndrome (PCOS)-treatment and control groups.

Class	Metabolite	Formula	Compound ID	p-value	ROC	Up or Down
13-HPODE	Eicosanoid	C18H32O	Com_246_neg	4.90E-08	1	down
5’-Adenylic acid	Nucleotide	C10H14N5O7P	Com_14_neg	2.72E-05	0.926667	up
D-Ribose 5-phosphate	Organic	C5H11O8P	Com_99_neg	5.84E-05	0.933333	up
Creatine	Organic	C4H9N3O2	Com_654_pos	0.000196	0.913333	up
Hypoxanthine	Nucleotide	C5H4N4O	Com_490_pos	0.000221	0.94	up
2-Phosphoglyceric acid	Carbohydrates	C3H7O7P	Com_257_neg	0.000248	0.9	up
Guanosine monophosphate	Nucleotide	C10H14N5O8P	Com_405_pos	0.000274	0.9	up
Lysopc 14:0	Phospholipid	C22H46NO7P	Com_327_pos	0.000305	0.88	up
N-Acetyl-L-leucine	Amino	C8H15NO3	Com_561_pos	0.000363	0.86	up
Pyrroloquinoline Quinone	Organoheterocyclic compounds	C14H6N2O8	Com_22_neg	0.000444	0.893333	up

### 3.5 Metabolite enrichment and metabolic pathway analysis

**Figure 5:**
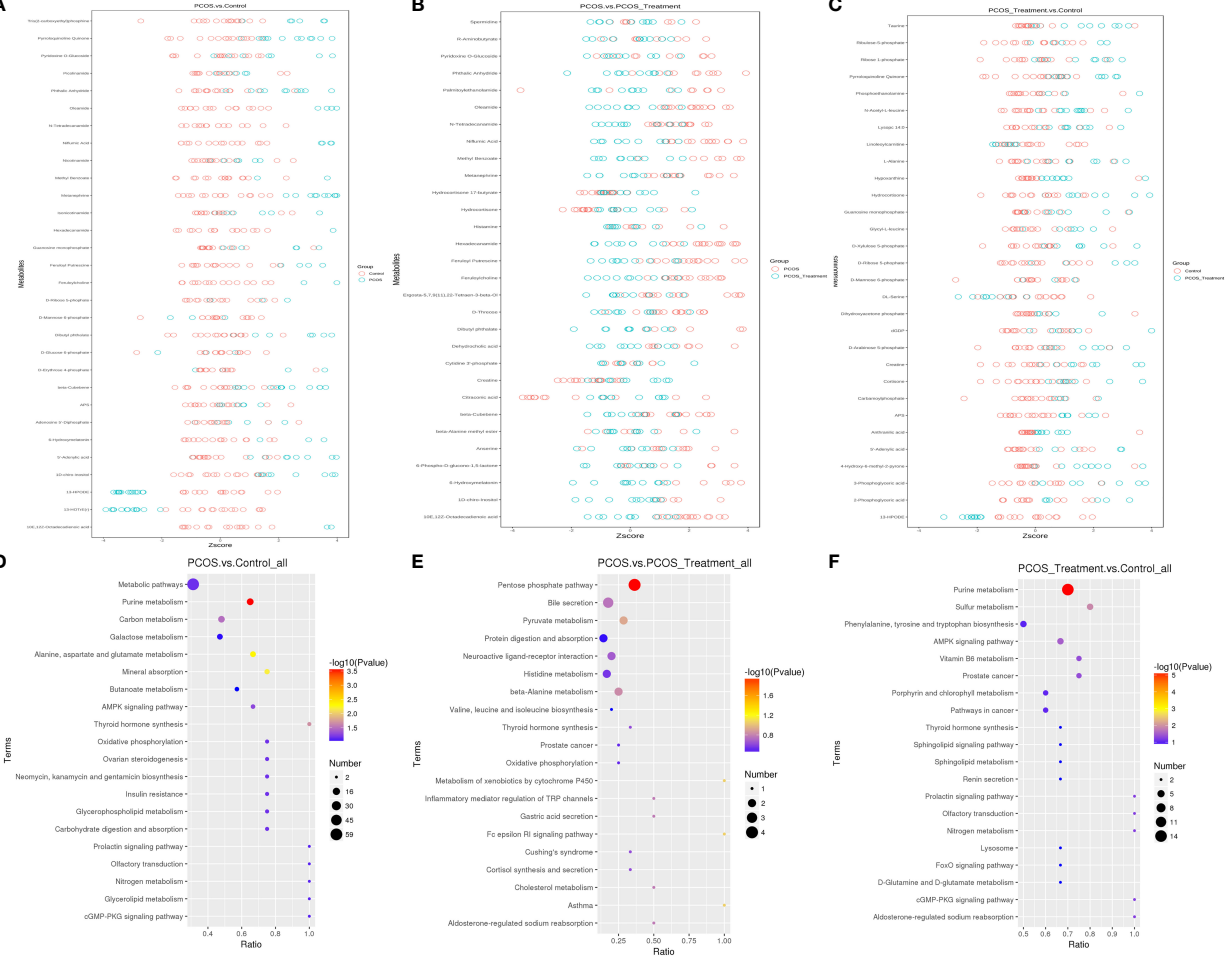
Z-scores and Kyoto Encyclopedia of Genes and Genomes (KEGG) analysis of differences in metabolites among different groups. **(A–C)** Z-score analysis of relative quantification values of metabolites on the same horizontal plane between different groups, Z-score values on the horizontal axis, and differential metabolites on the vertical axis. Only the top 30 metabolites (in descending order of P-values) are shown in the figure. **(D–F)** KEGG analysis of pathways enriched in differential metabolites between different groups. Pathway enrichment can determine the main biochemical metabolic and signal transduction pathways involved in differential metabolites. The horizontal axis in the figure is x/y (the number of differential metabolites in the corresponding metabolic pathway/the total number of metabolites identified in the pathway), and the larger the value, the higher the enrichment level of differential metabolites in the pathway. The colour of the dot represents the P-value value of the hypergeometric test, and the smaller the value, the greater the reliability and statistical significance of the test. The size of the point represents the number of differential metabolites in the corresponding pathway, and the larger the point, the more differential metabolites in the pathway.

The Z-score (standard score) is a value converted from the relative quantitative value of metabolites and is used to measure the relative quantitative value of metabolites on the same level. As shown in **Figures 5A–F**, metabolic pathways were significantly enriched when comparing PCOS and control groups, including tris (2-carboxyethyl) phosphine, pyrroloquinoline quinone, pyridoxine D-glucoside and picolinamide; when comparing the PCOS and PCOS-treatment groups, spermidine, R-aminobutyrate, pyridoxine O-glucoside, phthalic anhydride and palmitoylethanolamide were significantly enriched in metabolic pathways; taurine, ribulose-5-phosphate, ribose-1-phosphate, pyrroloquinoline quinone and phosphoethanolamine were significantly enriched between the PCOS-treatment and control groups.

The KEGG is a powerful tool for conducting metabolic analysis and metabolic network research in organisms. The enrichment results are expressed in the KEGG pathway, based on the principle of the hypergeometric distribution test. The results showed that purine metabolism, alanine, aspartate and glutamate metabolism, mineral absorption, thyroid hormone synthesis and carbon metabolism were significantly enriched metabolic pathways when comparing PCOS vs control groups. Pentose phosphate pathways, bile secretion and pyruvate metabolism were significantly enriched metabolic pathways when comparing PCOS vs PCOS-treatment groups. In addition, the first three significantly enriched KEGG pathways for metabolites between the PCOS-treatment and control groups included purine metabolism, sulphur metabolism, and phenylalanine, tyrosine and tryptophan biosynthesis.

## 4 Discussion

In addition to causing reproductive dysfunction, such as infertility, pregnancy complications, and risk of miscarriage, PCOS is closely related to metabolic abnormalities, such as IR, hyperinsulinemia, cardiovascular and cerebrovascular diseases, and lipid metabolism disorders, which can seriously affect quality of life (4, 14). In this study, we assessed the differences in serum metabolomics between patients with PCOS and healthy controls and observed the changes in serum metabolomics again after 3 months of COC treatment in patients with PCOS. The metabolomics analysis showed that, after COC treatment, there were significant changes in the serum metabolomics of patients with PCOS, and their composition was closer to that of the healthy control group.

COC has a reducing effect on free testosterone levels and can significantly block peripheral androgen levels (15, 16). The continuous decrease in high androgen production levels is achieved through feedback from the hypothalamic–pituitary axis, which is beneficial for increasing sensitivity to clomiphene and ultimately achieving the goal of improving patients’ sex hormone levels (17). Recent studies have found that hyperandrogenism plays a significant role in gut microbiota dysbiosis, and changes in gut microbiota can affect changes in serum testosterone levels, alter metabolomics, and promote insulin resistance. The results of this study showed that patients with PCOS who received COC treatment had a significant improvement in their serum sex hormone levels, a reduction in LH levels, and a significant reduction in the levels of biologically active free testosterone in the blood.

Metabolomics excavates biologically meaningful information from complex data structures through the collection, pre-processing and data analysis of biological samples, providing biological explanations and solving clinical problems (18, 19). The development and transformation application of metabolomics technology provides a new platform for constructing diagnostic and treatment strategies for PCOS, in addition to exploring its pathogenesis (20, 21). The results of this study showed that, following COC treatment, there were significant changes in the serum metabolic components of patients with PCOS. The differential metabolite correlation analysis in patients with PCOS and healthy controls revealed significant differences in several metabolites, including N-tetradecanamide, hexadecanamide, 10E, 12Z-octadecadienoic acid and 13-HOTrE(r); after 3 months of COC treatment, significant differences in benzoic acid, organic acid and phenolamides were observed. The long-chain fatty acids involved in fatty acid metabolism (represented by 12Z octadecadienoic acid) were higher in the PCOS group, indicating an increase in long-chain fatty acids and enhanced lipolysis of adipose tissue in PCOS. After COC treatment, organic acids decreased, suggesting that COC may inhibit fatty acid synthesis and enhance fatty acid metabolism, improving lipid metabolism disorders in patients with polycystic ovary syndrome (22). In addition, the decrease in serum 13-HODE and 13-HOTrE(r) suggested a reduction in the patient’s oxidation level and a significant improvement in treatment effectiveness (23). The mechanism of action of COCs is on androgen receptors, promoting the generation of liver sex hormone binding globulin, which is closely related to IR and lipid metabolism, among other processes (24).

Using GC–MS to analyse blood sera in each group, the characteristic changes seen in PCOS were metabolic disorders of amino acids, carbohydrates, and purines, with the most significant changes in total cholesterol, uric acid, phenylalanine, aspartic acid, and glutamate (25–27). In addition, studies have confirmed that high levels of uric acid in healthy women of childbearing age can lead to an increased risk of anovulation and cardiovascular disease. Based on this speculation, the increase in uric acid levels may be related to ovulation dysfunction in PCOS, which elucidates the mechanism of ovulation dysfunction in PCOS from another perspective (26, 28, 29). In addition, in myocardial cells, high uric acid can lead to insulin resistance and inhibition of glucose metabolism, which may be the cause of long-term cardiovascular disease in patients with PCOS (30). Elevated serum uric acid levels are a common metabolic abnormality in PCOS, therefore, early prevention and treatment of PCOS are of great significance. However, whether improving uric acid levels can improve metabolic abnormalities in PCOS deserves further exploration in the future. The decrease in phenylalanine, glutamic acid, and aspartic acid can be used to predict whether patients with PCOS will experience impaired glucose tolerance and type 2 diabetes and can also provide information on the pathogenesis of PCOS and impaired glucose tolerance/diabetes (31). Following COC treatment, the above metabolic disorder was found to be significantly alleviated.

Thus far, metabolomics has flourished and has made outstanding contributions to the development of the medical field. The data obtained from metabolomics are complex and diverse, which requires rigorous and meticulous data processing. As for the current metabolomics-based research on PCOS, the overall sample size was relatively small. Therefore, subsequent research should expand sample sizes, use strictly designed experimental plans, and search for the best experimental samples and metabolomics techniques in order to achieve early and accurate diagnosis of PCOS, adopt effective treatment plans, and prevent long-term complications.

In conclusion, this study showed the improvements of COC treatment on sex hormone levels, endocrine factors, and metabolite levels in patients with PCOS, indicating that the use of COCs had a significant effect on the treatment of PCOS. Therefore, COC treatment for patients with PCOS is worthy of clinical promotion.

## Data availability statement

The datasets presented in this study can be found in online repositories. The names of the repository/repositories and accession number(s) can be found in the article/supplementary material.

## Ethics statement

The studies involving humans were approved by Ethical Research Committee at the First People’s Hospital of Yunnan Province (approval number: 2018FE117). The studies were conducted in accordance with the local legislation and institutional requirements. Written informed consent for participation in this study was provided by the participants' legal guardians/next of kin.

## Author contributions

TZ: Data curation, Investigation, Validation, Writing – original draft, Writing – review & editing. XX: Data curation, Investigation, Project administration, Writing – original draft. LL: Conceptualization, Data curation, Writing – original draft. JZ: Formal analysis, Investigation, Resources, Writing – original draft, Writing – review & editing. WH: Conceptualization, Data curation, Writing – original draft, Writing – review & editing. QZ: Data curation, Investigation, Writing – original draft, Writing – review & editing. JW: Conceptualization, Formal analysis, Supervision, Writing – original draft. XW: Conceptualization, Data curation, Methodology, Software, Writing – original draft. TY: Conceptualization, Formal analysis, Funding acquisition, Methodology, Writing – original draft.
